# Metabolic Regulation of Group 3 Innate Lymphoid Cells and Their Role in Inflammatory Bowel Disease

**DOI:** 10.3389/fimmu.2020.580467

**Published:** 2020-10-26

**Authors:** Dongjuan Song, Lijie Lai, Zhihua Ran

**Affiliations:** Division of Gastroenterology and Hepatology, Key Laboratory of Gastroenterology and Hepatology, Ministry of Health, Inflammatory Bowel Disease Research Center, Renji Hospital, School of Medicine, Shanghai Jiao Tong University, Shanghai Institute of Digestive Disease, Shanghai, China

**Keywords:** group 3 innate lymphoid cells, immunometabolism, intestinal inflammation, inflammatory bowel disease, therapeutics

## Abstract

Inflammatory bowel disease (IBD) is characterized by chronic and relapsing inflammatory disorder of the intestine. IBD is associated with complex pathogenesis, and considerable data suggest that innate lymphoid cells contribute to the development and progression of the condition. Group 3 innate lymphoid cells (ILC3s) not only play a protective role in maintaining intestinal homeostasis and gut barrier function, but also a pathogenic role in intestinal inflammation. ILC3s can sense environmental and host-derived signals and combine these cues to modulate cell expansion, migration and function, and transmit information to the broader immune system. Herein, we review current knowledge of how ILC3s can be regulated by dietary nutrients, microbiota and their metabolites, as well as other metabolites. In addition, we describe the phenotypic and functional alterations of ILC3s in IBD and discuss the therapeutic potential of ILC3s in the treatment of IBD.

## Introduction

Inflammatory bowel disease (IBD) is a group of chronic inflammatory conditions of the bowel comprising ulcerative colitis (UC) and Crohn’s disease (CD). The increased incidence and prevalence of IBD in recent years poses a significant challenge to society (1, 2). Mounting evidence suggests that genetic background, environmental factors, diet, microbiotic dysbiosis and immune dysregulation contribute to the initiation and progression of IBD (3). Innate lymphoid cells (ILCs) belong to the same family as lymphocytes, however, they lack the rearranged antigen receptors expressed by T cells and B cells and play a central role in immunity, inflammation and gut barrier function (4, 5). The ILC family is classified into three groups: group 1 ILCs including natural killer (NK) cells and ILC1s, group 2 ILCs (ILC2s), and group 3 ILCs (ILC3s). Group 1 ILCs require the transcription factor T-bet and secrete interferon gamma (IFN-γ) upon stimulation with interleukin (IL)-12, IL-15 and IL-18. ILC2s require the transcription factor GATA3 and produce IL-4, IL-5 and IL-13 upon stimulation with IL-25 and IL-33. ILC3s express IL-22, IL-17 and granulocyte macrophage colony-stimulating factor (GM-CSF) when stimulated with IL-23 and IL-1β, which depends on RAR-related orphan receptor gamma t (RORγt) and arylhydrocarbon receptor (AHR) (6, 7). In addition, regulatory ILCs (ILCregs), a novel regulatory subset of ILCs, can produce IL-10 and transforming growth factor β1 (TGF-β1) and help to decrease intestinal inflammation (8).

Single cell analysis of ILC subsets in the small intestine of mice revealed that genes expressed by ILC3s were highly enriched for carbohydrate metabolism and glycolysis, which is different from ILC1s and ILC2s and indicates that each subset of ILC has a unique metabolic profile (9). Another study demonstrated that ILC3 activation relies on the mTOR complex 1(mTORC1)-hypoxia-inducible factor 1α (HIF1α) pathway, which promotes glycolysis and RORγt expression, to promote cellular proliferation as well as IL-22 and IL-17A production. Meanwhile, mTORC1 signaling in ILC3s can activate mitochondrial metabolism and the production of mitochondrial ROS (mROS), which prolongs HIF1α activity, promotes RORγt expression, and ultimately facilitates ILC3 activation. Briefly, ILC3s utilize both glycolysis and mROS production to support effector function (10). ILC3s are enriched in gut mucosal tissue and have a specialized capacity to sense multiple exogenous and endogenous signals, and function as “communication hubs” of the intestinal immune system (11). Indeed, signals from nutrient-derived metabolites, microbiota and microbial metabolites as well as other host metabolites such as 7a,25-dihydroxycholesterol and prostaglandin E2 can be interpreted by ILC3s to regulate proliferation, migration and function of ILC3s as well as their interactions with other cells, which is vital for tissue homeostasis. Dysregulation of ILC3s has been implicated in the pathogenesis of IBD and colorectal cancer (6, 12–14). A better understanding of ILC3s biology in patients with IBD provides valuable insights into potential therapeutic targets. Therefore, it is necessary to evaluate the environmental cues that activate and suppress ILC3s in the gut. In this review, we discuss recent work on how ILC3s are regulated by environmental cues and summarize the involvement of ILC3s in IBD as well as their potential application in IBD therapy.

## Overview of Group 3 Innate Lymphoid Cells

ILC3s are a heterogeneous group of cells in humans and mice. Single-cell sequencing analyses of ILCs in human tonsils revealed at least three subsets of ILC3s based on the expression of NKp44, human leucocyte antigen D-related (HLA-DR), and CD62L (15). Furthermore, human NKp44^+^ILC3s are enriched in barrier tissues such as colon and skin (16). MHCII^+^ ILC3s with antigen presenting function have also been identified in the colon and small intestine in humans and mice (17, 18). In addition, single-cell sequencing analyses of CD127^+^ILCs from the small intestinal lamina propria of mice identified five transcriptional states of ILC3s (9). In mice, ILC3s can be divided into CCR6^+^T-bet^-^ and T-bet^+^ILC3s (19). CCR6^+^ILC3s contain fetal lymphoid tissue inducer (LTi) cells, which are indispensable for the organogenesis of secondary lymphoid organs (20). Adult CCR6^+^LTi-like ILC3s have similar phenotypes to fetal LTi cells and are essential for the development of cryptopatches (CPs) and isolated lymphoid follicles (ILFs) in the gut (21). Although both fetal LTi and adult LTi-like ILC3 express RORγt, they develop from a progenitor distinct from all other ILC subsets (22). T-bet^+^ILC3s can be further classified on the basis of the expression of natural cytotoxicity receptor (NCR) (NKp46 in mice). T-bet plays a critical role in the differentiation of NCR^+^ ILC3 from its NCR^-^ILC3 precursors as well as IFN-r and IL-22 production in NCR^+^ ILC3 (23, 24).

## Regulation of ILC3s by Dietary Nutrients

Recent evidence indicates that dietary vitamins function as key modulators of ILC3s biology (**Table 1**). For instance, mice fed a diet deficient in vitamin D exhibit reduced abundance of ILC3s and IL-22 secretion by colonic ILC3s, leading to increased susceptibility to *Citrobacter rodentium* (*C. rodentium*) infection (25). Consistently, global deletion of vitamin D receptor (VDR) or deficiency in VDR ligand in mice leads to reduced colonic ILC3s and impaired ILC3 response, leading to increased susceptibility to bacterial infection compared with wild-type mice (26). Furthermore, *in vitro* and *in vivo* studies revealed that vitamin D/VDR signaling can stimulate colonic ILC3 proliferation, especially LTi cells (26). In contrast, another study reported that VDR knockout (KO) mice had enhanced resistance to bacterial infection due to increased frequencies of ILC3s in the gut and enhanced expression of IL-22 as well as anti-bacterial peptides (39). Of note, an *in vitro* study revealed that 1α, 25-dihydroxy vitamin D3 (1,25D3), the active form of vitamin D, downregulates the IL-23 receptor pathway in human NKp44^+^ ILC3s, inhibiting IL-22 and GM-CSF production and inversely enhancing IL-6 production, which encourages ILC3s to maintain an innate-like cytokine expression profile (27). Consequently, these results suggest vitamin D is not only necessary for development and function of ILC3s at steady state, but also can restrain the pro-inflammatory properties of ILC3s.

**Table 1:** Metabolic regulation of ILC3s by nutrient-derived metabolites.

Nutrients	Function	Species	Refs
Vitamin D	Promotes ILC3s population in both small intestine and colon Regulates IL-22 production in colonic ILC3s	Mouse *In vivo*	(25)
1,25D3	Modulates frequency of CD3^-^RORγt^+^ILC3s in colon, mainly LTi cells Regulates IL-22 production in ILC3s Regulates colonic ILC3s expansion, mainly LTi cells	Mouse *In vivo* and *in vitro*	(26)
	Inhibits IL-22 and GM-CSF production, whereas enhances IL-6 production in activated Nkp44^+^ILC3s	Human *In vitro*	(27)
	Antagonizes α4β7 expression in human ILC3s induced by RA and IL-2	Human *In vitro*	(28)
Vitamin A Retinoic acid	Promotes ILC3s population and function Controls a proliferative balance between ILC3s and ILC2s Controls formation of solitary intestinal lymphoid tissue postnatally Regulates the postnatal differentiation of intestinal ILC3s	Mouse *In vivo* and *in vitro*	(29–31),
	Regulates homing receptor switch in ILC3s, and thereby regulates the migration of ILC3s to the gut	Mouse and human *In vivo* and *in vitro*	(28, 32),
	Accelerates the differentiation of human ILC1s to IL-22-producing ILC3s driven by IL-2, IL-1β and IL-23	Human *In vitro*	(33)
Dietary AHR ligands	Modulates postanal expansion of CD4^-^ILC3s Controls the formation of intestinal lymphoid follicles Regulates IL-22 production in ILC3s Modulates ILC3s population	Mouse *In vivo*	(34–36),
Maternal retinoids	Controls fetal CD4^+^LTi cells differentiation via RORγt Controls the size of secondary lymphoid organs Determines the immune fitness in adult offspring	Mouse *In vivo*	(20)
Maternal high-fat diet	Increases IL-17-producing NKp46^+^ILC3s in the lamina propria of offspring	Mouse *In vivo*	(37)
Maternal TCDD	Reduces colonic ILC3s population and expression of RORγt in colonic ILC3s, and increases the frequency of colonic NKp46^+^ILC3 in offspring	Mouse *In vivo*	(38)

Refs, references; 1,25D3,1α, 25-dihydroxy vitamin D3; GM-CSF, granulocyte macrophage colony-stimulating factor; RA, retinoic acid; AHR, aryl hydrocarbon receptor; RORγt, RAR-related orphan receptor gamma t.

Similar to vitamin D, lack of vitamin A in the diet results in reduced numbers of ILC3s, IL-22-producing ILCs, CPs and ILFs (29, 30) as well as decreased expression of CCR9 and α4β7 by ILC3s and ILC1s (32) in the small intestine of mice. Furthermore, retinoic acid (RA), a vitamin A metabolite, can induce a homing receptor switch in ILC3s from CCR7 to CCR9 and α4β7 leading to migration of ILC3s to the gut in mice (32) and enhance IL-22 secretion by ILC3s in the mouse small intestine during intestinal inflammation (31). In line with the findings in mice, RA works synergistically with IL-2 to induce integrin α4β7 expression in human ILC3s *in vitro*. Interestingly, 1,25D3 antagonizes α4β7 expression in human ILC3s induced by RA and IL-2, suggesting the biologically active metabolites of vitamin A and D have antagonistic effects on the expression of integrin in human ILC3s (28). Moreover, RA can accelerate the differentiation of human CD127^+^ILC1s into IL-22-producing ILC3s in the presence of IL-2, IL-1β and IL-23 (33).

The aryl hydrocarbon receptor (AHR), which is expressed by ILCs and other immune cells, can sense ligands generated from diet, microbiota metabolism, cellular metabolism and environmental pollutants (21). AHR mediates the regulatory effects of dietary and microbial metabolites on ILC3s. AHR-deficient mice exhibit increased vulnerability to colitis and intestinal *C. rodentium* infection, which is mainly due to the impaired accumulation of ILC3s and IL-22 production in the gut (34, 40, 41) and a decrease in numbers of intraepithelial lymphocytes (IELs) (42). Studies in mice have demonstrated that AHR is indispensable for intestinal ILC3s maintenance and function as well as postnatal development of intestinal lymphoid tissues (34, 40, 41). For instance, AHR promotes the survival of murine intestinal ILC3s, cooperates with RORγt to enhance IL-22 expression and facilitates the expression of IL-7 in the intestine and the expression of IL-7 receptor (IL-7R) by murine ILC3s (40). This is in keeping with the effect of IL-7/IL-7R signaling in the maintenance of ILC3s (43). Moreover, AHR facilitates the expansion of murine CD4^-^ILC3s through stimulation of cell proliferation, which is induced by AHR-controlled transcription of Kit (34). Additionally, AHR promotes the development of NCR^+^ILC3s and LTi-like ILC3s by, and partially through activation of Notch signaling (41). Notch signaling has been shown to be essential for adult ILC3s differentiation, but not for fetal LTi development (44). Notably, AHR is also known to block human IL-1R1^+^ILC3s differentiation into cytolytic NK cells (45).

The diet provides several exogenous and endogenous AHR ligands or precursors, such as Indole-3-Carbinol (I3C), natural flavonoids, resveratrol, curcumin and tryptophan (46, 47). The absence of dietary AHR ligands increases bacterial load or translocation and aggravates dextran sulfate sodium (DSS)-induced colitis and *C. rodentium* infection in mice (35, 42). The phenotype of mice fed with phytochemical-free diets mimics that of AHR-deficient mice, which have decreased numbers of ILC3s, insufficient postnatal proliferation of CD4^-^ILC3s, reduced IL-22 production, and impaired development of intestinal lymphoid follicles (**Table 1**). The addition of I3C to the diet can counteract these abnormalities (34, 36). Consistently, metabolic clearance of natural AHR ligands by constitutive *Cyp1a1* expression in mice contributes to loss of ILC3s and Th17 cells as well as reduced IL-22 production, further increasing vulnerability to *C. rodentium* infection (48). More importantly, dietary supplementation of AHR ligands or precursors ameliorates DSS-induced colitis and intestinal infection in mice (35, 48–50).

Indeed, several studies showed that ILC3 development can be regulated by maternal nutritional status (**Table 1**). Maternal levels of dietary retinoids are important for regulation of LTi cells differentiation, for ensuring the correct size of secondary lymphoid organs and for maintenance of immune fitness in adult offspring in mice (20). In addition, a maternal high-fat diet (HFD) has been confirmed to induce the expansion of IL-17-producing NKp46^+^ILC3s in mice offspring, dependent on the subsequent microbiota alterations (37). Moreover, exposure to TCDD (a ligand of AHR) during pregnancy and lactation in mice led to reduced frequency of colonic ILC3s and decreased expression of RORγt in colonic ILC3s, and increased frequency of colonic NKp46^+^ILC3 in offspring (38). These results suggest that environment cues in pregnancy can modulate ILC3 biology in offspring.

Taken together, these findings suggest that host nutritional status can regulate the numbers and function of ILC3s, immune response and susceptibility to colitis, thus dietary refinements and nutrient supplementation may be beneficial in alleviating the severity of IBD.

## Regulation of ILC3s by Microbiota and Microbial Metabolites

**Figure 1:**
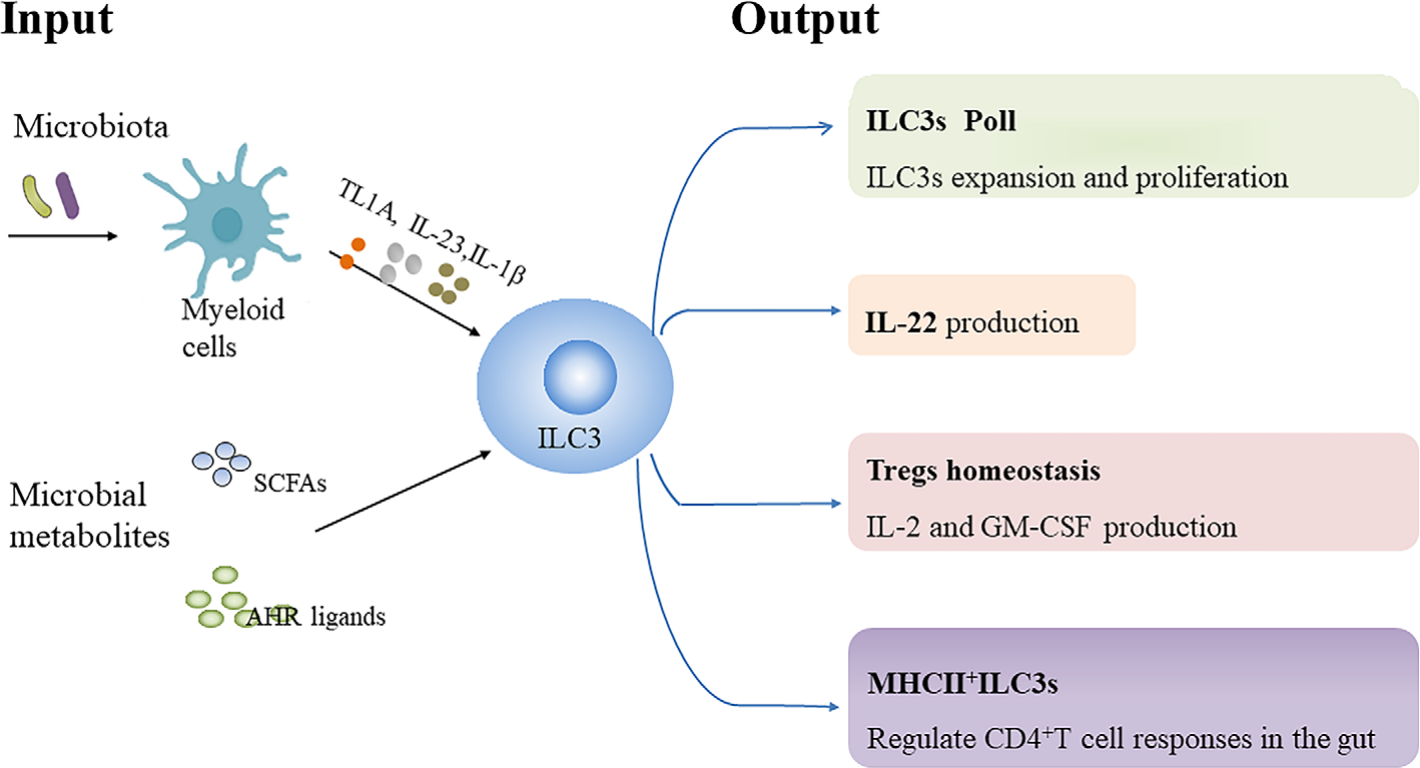
Regulation of ILC3s by microbiota and microbial metabolites. Microbiota-driven IL-1β production by intestinal macrophages enhances IL-2 and granulocyte macrophage colony-stimulating factor (GM-CSF) production by ILC3s, which is essential for regulatory T cells (Tregs) and immunologic homeostasis in the small intestine and colon in mice, respectively. Microbiota-induced IL-23 reduces major histocompatibility complex class II (MHCII) expression in murine NCR^-^ILC3s, thereby negatively affecting their potential to elicit CD4^+^T cell responses. TNF-like ligand 1 A (TL1A) cooperates with IL-23 and IL-1β to promote IL-22 production, proliferation and expansion of human ILC3s *ex vivo*. Additionally, TL1A can enhance IL-22 secretion by murine ILC3s. However, TL1A expression can induce OX40L expression in MHCII^+^ILC3s, which promote Th1 cells activation in chronic T cell colitis in mice. Collectively, microbial signals can be sensed by myeloid cells to regulate ILC3s biology. In addition, Short-chain fatty acids (SCFAs), the metabolites from gut microbial fermentation of dietary substrates, can regulate murine ILC3s pool as well as their IL-22 production in a receptor-dependent manner. Dietary tryptophan can be metabolized into aryl hydrocarbon receptor (AHR) ligands by commensal microbiota, which promote IL-22 secretion by murine ILC3s.

Commensal flora has been demonstrated to promote or repress the function or differentiation of NKp46^+^ILC3s in mice (51–53). However, the development of murine ILC3s seems to be programmed independent of the gut microbiota (41, 53, 54). Moreover, human ILC3s from tissue exposed to the fecal stream produce more IL-22 compared with ILC3s from that not exposed, indicating that the function of human ILC3s is influenced by microbiota (55). Herein, we review the influence of microbiota and microbial metabolites on ILC3s biology (**Figure 1**).

The production of IL-22 rather than IFN-r by human colonic ILC3s is significantly enhanced following stimulation with commensal or pathogenic bacteria, which may be mediated indirectly by IL-23-and IL-1β-producing CD11c^+^ myeloid dendritic cells (mDC) and ligation of the NKp44 receptor (56). Additionally, microbiota can stimulate IL-1β production by macrophages through the MyD88 and Nod2 pathway to promote IL-2 production in murine ILC3, which is essential for maintaining Tregs and immunologic homeostasis in the small intestine in mice (57). Moreover, Microbiota-driven IL-1β secretion by intestinal macrophages enhances GM-CSF production by ILC3s, which in turn regulates the frequency and function of macrophages and dendritic cells, helping to maintain colonic Tregs homeostasis (58) (**Table 2**). Notably, microbiota-induced IL-23 can reduce MHCII expression through mTORC1 and STAT3 signaling in NCR^-^ ILC3s in the small intestine of mice, thereby negatively affecting their potential to induce CD4^+^ T-cell immune responses (18).Taken together, these findings suggest that microbial signals can be sensed by myeloid cells to regulate ILC3s function.

**Table 2:** The dichotomous role of ILC3-derived cytokines in the intestinal immune response.

Cytokine	Protective	Pathogenic
**IL-22**	Promotes epithelial regeneration, proliferation and glycosylation (59–61),	Contributes to the development of acute innate colitis in mice (62)
	Promotes production of anti-bacterial peptides and mucins (63)	Induces endoplasmic reticulum stress (ER) in colonic epithelial cells (64)
	Protects intestinal stem cells from genotoxic stress, limiting tumorigenesis (36)	Increases the risk of colitis-associated cancer (14, 65, 66),
		Participates in intestinal fibrosis (67, 68),
**GM-CSF**	Maintains colonic Tregs homeostasis and intestinal homeostasis (58) Suppresses wound-healing, pro-fibrotic macrophage phenotype, reduces progression to intestinal fibrosis (69)	Mobilizes ILC3s from cryptopatches into adjacent tissue (70) Promotes accumulation of inflammatory monocytes in the intestine (70, 71),. Regulates intestinal macrophage polarization, drives pro-inflammatory macrophage phenotype (69).
**IL-17**		Participates in intestinal fibrosis (68)
		Contributes to colitis development in TRUC mice (72) and bacteria-driven innate colitis in mice (73)
**IFN-r**	Controls production of mucin and protects the epithelial barrier against salmonella infection (23)	Contributes to colitis development in bacteria-driven innate colitis in mice (73)

GM-CSF, granulocyte macrophage colony-stimulating factor; IFN-r: interferon-r.

Tumor necrosis factor superfamily member 15 (TNFSF15) has been identified as a susceptibility gene for CD and is associated with the adaptive immune response (74). The TNFSF15 gene encodes TNF-like ligand 1 A (TL1A) protein, which is the ligand for death domain receptor 3 (DR3). Although TL1A supplementation alone does not increase IL-22 production and proliferation of human ILC3s, TL1A cooperates with IL-23 and IL-1β to promote IL-22 production, proliferation and expansion of human ILC3s *ex vivo* (55, 75). Colonization of mice with adherent IBD-associated microbiota such as segmented filamentous bacteria (SFB) and adherent-invasive *Escherichia coli* (AIEC) strain 2A enhance IL-22 production by ILC3s (76, 77). Further evidence revealed firstly that SFB and AIEC strain 2A facilitate TL1A expression from Cx3cr1^+^ mononuclear phagocytes (MNPs) and secondly that TL1A enhances IL-22 production in murine ILC3s and protects mice against acute colitis, which is dependent on DR3 expressed by ILC3s. Mice with ILC3s-specific DR3 deletion show defective IL-22 secretion by ILC3s and increased susceptibility to DSS-induced colitis (78). Nevertheless, activation of DR3 contributes to the reduced abundance of ILC3s in a GM-CSF-and IL-23-dependent manner, which consequently exacerbates DSS-induced colitis in mice (79). OX40L expression by ILC3s has been demonstrated to be essential for homeostatic expansion of intestinal regulatory T cells (Tregs) in mice, and the expression of OX40L can be increased by TL1A and viral stimulation or inhibited by CD4^+^ T cells (80). However, TL1A-induced OX40L expression on MHCII^+^ILC3s promotes the activation of T-BET^+^ Th1 cells, which is essential for chronic T-cell colitis in mice (78). Additionally, neutralization of TL1A can attenuate α-CD40-induced colitis and DSS-induced chronic colitis in mice (79, 81). Taken together, these data suggest that TL1A/DR3 signaling may help to maintain mucosal homeostasis and protect against acute injury, but play a detrimental role in chronic intestinal inflammation.

Short chain fatty acids (SCFAs), the metabolites from gut microbial fermentation of dietary substrates, exert modulating effects on immune cells and provide energy support for intestinal epithelial cells (IECs) (82). “Metabolite-sensing” free fatty acid receptor 2 (FFAR2/GPR43), also known as a receptor for SCFAs, is expressed by colonic ILC3s. Acetate and propionate (the natural FFAR2 ligands) and synthetic FFAR2 agonists contribute to the proliferation of colonic ILC3s and the production of IL-22 in ILC3s from mice, subsequently contributing to host defenses against DSS-induced colonic injury and *C. rodentium* infection in mice (83). Further evidence revealed that acetate facilitates IL-22 production in murine ILC3s upon stimulation with IL-1β *via* enhancement of IL-1 receptor expression in a FFAR2-dependent fashion (84). Of note, butyrate inhibits the number of NKp46^+^ILC3s as well as their IL-22 production in mouse terminal ileal Peyer’s patches (PPs) through the GPR109a receptor under steady-state conditions, which leads to a reduced frequency of Tregs and antigen-specific immune induction in terminal ileal PPs (85). In accordance with this, *Gpr109a^-/-^Rag1^-/-^* mice developed spontaneous colonic inflammation and had increased ILC3s in the gut relative to *Rag1^-/-^* mice especially IL-17-producing ILC3s. Mechanistically, GPR109a suppresses ILC3s through inhibiting microbiota-induced IL-23 production in intestinal dendritic cells to regulate intestinal homeostasis (86). Taken together, the regulation of SCFAs on ILC3 responses may depend on subset, receptor or tissue environment as well as host conditions.

Tryptophan (Trp) metabolites from symbiotic microbiota can promote epithelial barrier function and inhibit the inflammatory response (87–89). *Lactobacillus reuteri* (*L. reuteri*) can metabolize Trp into endogenous AHR ligands such as indole-3-aldehyde (IAID) or indole-3-lactic acid under conditions of Trp sufficiency, which promotes IL-22 secretion by ILC3s and induces gut intraepithelial CD4^+^CD8αα^+^ T cells in an AHR-dependent manner in mice (87, 90). The microbiota from mice lacking caspase recruitment domain family member 9 (CARD9), a susceptibility gene for IBD, fail to metabolize tryptophan into AHR ligands, resulting in decreased production of IL-22 by ILC3s and Th22 cells, and increased susceptibility in germ-free recipients to colitis (91). Importantly, impaired AHR activation and metabolism of tryptophan by the gut microbiota has been identified in patients with IBD, which is associated with a CARD9 phenotype (91). Administration of some strains of *L.* *reuteri* alone or in combination with other *Lactobacillus* strains has been reported to prevent DSS-induced colitis in mice due to various mechanisms (91–94) and some studies suggest that AHR activation and enhanced IL-22 production play a critical role in the process (91, 92). In addition, it has been observed that rectal administration of *L. reuteri* can attenuate mucosal inflammation in children with active distal UC (95). Nevertheless*, L. reuteri* can attenuate immune checkpoint blockade-associated colitis through reducing the population of ILC3s (96).

Apart from bacterial tryptophan metabolism, tryptophan in the gastrointestinal tract can also be metabolized through the kynurenine pathway *via* the rate-limiting enzyme indoleamine 2,3 dioxygenase-1 (IDO1) and the serotonin pathway *via* Trp hydroxylase 1 (TpH1) (97). Increased IDO1 expression in the gut negatively correlates with the number of ILC3s as well as IL-17 and TNF-a production by ILC3s during simian immunodeficiency virus infection (98). Interestingly, IDO1-knockout mice had increased numbers of ILC3s in the lungs compared with wild-type mice after *paracoccidioides brasiliensis* infection (99). Deficiency or inhibition of IDO1 aggravates 2,4,6-trinitrobenzene sulfate (TNBS)-induced colitis in mice (100, 101). In contrast, IDO1-knockout mice are less susceptible to DSS-induced colitis (102). Mice lacking IDO1 had increased abundance of bacteria with tryptophanase activity, which results in the accumulation of microbiota-derived AHR ligands (87, 102). Thus, endogenous Trp metabolism may influence the gut microbiota and bacterial Trp metabolism, thereby regulating the innate lymphoid cells.

## Regulation of Other Metabolites

G-Protein-Coupled Receptor 183 (GPR183) and its ligand 7a,25-dihydroxycholesterol (7a,25-OHC), a hydroxylated metabolite of cholesterol, are critical for modulating the distribution of ILC3s, and subsequent interactions between ILC3s and T follicular helper cells (TfH) and B cells (103–105). The GPR183 receptor expressed on LTi-like ILC3s can sense 7a,25-OHC that is produced from fibroblastic stromal cells located in lymphoid structures, which contributes to ILC3s migration to CPs and ILFs and the formation of colonic lymphoid tissues at steady state in mice (103). In addition, GPR183 and 7a,25-OHC regulate the distribution or accumulation of ILC3s in the mesenteric lymph nodes (mLNs), PPs and small intestine of mice (104, 105). ILC3s located within the interfollicular border of mLNs limit TfH-driven B cell responses and IgA production through antigen presentation in the colon at steady state in mice, which is beneficial for the maintenance of host-microbiota mutualism (105). Moreover, GPR183 can protect mice against *C. rodentium* infection through promoting the enrichment of IL-22-expressing ILC3s in the small intestine of mice (104). However, mice treated with CD40 Ab had enhanced 7a,25-OHC production, which in turn promotes colitis through activating the migration of GPR183^+^ ILC3s and myeloid cells to inflammatory foci (103). In addition, prostaglandin E2 (PGE2) promotes homeostasis and functionality of murine ILC3s *via* its receptor EP4, leading to the inhibition of systemic inflammation in mice (106). Adenosine 5′-triphosphate (eATP) and its metabolite adenosine inversely regulate IL-22 secretion from murine ILC3s. Inhibition of NTPDases, which hydrolyzes extracellular eATP into adenosine, can aggravate DSS-induced colitis in mice dependent on reduced frequency of IL-22-producing ILC3s (107).

## ILC3s in IBD

Decreased frequency of NKp44^+^ILC3s has been observed in inflamed tissue from IBD patients compared with non-IBD controls, which was related to disease severity regardless of whether patients were newly diagnosed or had established disease. However, the frequency of ILC1s and ILC2s was increased in newly-diagnosed CD and UC, respectively, and the frequency of ILC1s and ILC2s were both increased in patients with established IBD (12). Reduced frequency of NKp44^+^ ILC3s in inflamed intestinal tissues from CD patients was accompanied by enrichment of IFN-γ-secreting CD127^+^ILC1s, indicating that an imbalance between ILC3 and ILC1 may contribute to the pathogenesis of CD (33, 108). Furthermore, the frequency of NKp44^+^ ILC3s was shown to have an inverse association with the accumulation of IL-17A^+^ IFN-γ^+^ and IL-22^+^IFN-γ^+^ T cells in inflamed regions of adults with CD (13). In addition, MHCII^+^ILC3s were significantly reduced in inflamed regions of CD patients compared with non-inflamed regions (13). MHCII^+^ ILC3s have been reported to mediate negative selection *via* antigen presentation together with IL-2 withdrawal, leading to cell death of activated commensal bacteria–specific T cells in mice (17). These results suggest that downregulation of MHCII expression may be associated with aberrant immune responses in IBD.

IL-22 functions as a dichotomous cytokine in intestinal inflammation (**Table 2**). IL-22 deficient mice exhibit aggravated experimental colitis following DSS exposure (109), and IL-22 orchestrates epithelial regeneration, proliferation and glycosylation, the production of mucins and anti-bacterial peptides and protects intestinal stem cells from genotoxic stress (36, 59–61, 63). NKp44^+^ILC3s produced less IL-22, but acquired the ability to secrete IFN-γ in the inflamed terminal ileum of CD patients compared with unaffected tissue (13). However, IL-22-expressing ILC3s have been confirmed to be responsible for the development of acute innate colitis in mice (62). Foxp3^+^ Treg cells can attenuate IL-22^+^ ILC3s-mediated colitis in mice through inhibiting the secretion of IL-23 and IL-1β by Cx3cr1^+^macrophages (110). Additionally, patients with active mild-to-moderate IBD had increased production of IL-22 in colonic ILC3s compared with controls (55). Serum IL-22 concentrations were markedly increased in CD patients compared with healthy volunteers, and positively correlated with disease activity (111). Furthermore, IL-22 can induce endoplasmic reticulum stress (ER) in colonic epithelial cells, which is functionally important in chronic colitis. Importantly, IL22-responsive transcripts and ER stress response modules were enriched in the colons of patients with IBD compared with non-IBD controls, and the IL22-responsive transcriptional modules positively correlated with the severity of mucosal injury (64). In addition, IL-22 may be implicated in intestinal fibrosis (67, 68). Increased expression of the IL-23/IL-22 axis regulated by mTOR/autophagy signaling in Cx3cr1^+^MNPs exacerbates fibrosis in the mouse model of TNBS-induced intestinal fibrosis. Interestingly, neutralization of either IL-23 or IL-22 can attenuate the fibrosis reaction. And ILCs, but not T and B cells, are likely participated in intestinal fibrosis (67).

The production of GM-CSF was elevated in LPMCs from the inflamed mucosa of patients with IBD compared with non-inflamed mucosa and non-IBD controls (112). In addition, the secretion of GM-CSF was also increased by blood ILCs in patients with IBD compared with healthy volunteers (70). Increased production of GM-CSF from ILC3s during colitis mobilizes the migration of ILC3s into adjacent tissue from cryptopatches and promotes inflammatory monocyte accumulation, which mediates the pathogenic role of ILC3s in anti-CD40-induced colitis in mice, and neutralization of GM-CSF can ameliorate intestinal colitis in mice (70, 71) (**Table 2**). However, GM-CSF gene knockout mice are more susceptible to acute DSS-induced colitis compared with wild-type mice (113). Importantly, elevated levels of GM-CSF auto-antibodies (Ab), which are associated with reduced GM-CSF bioactivity, have been proven to be associated with stricturing/penetrating behavior and higher incidence of intestinal resection in CD patients (114), surgical recurrence in ileal CD patients (115) and disease relapse in IBD patients (116). Importantly, sargramostim (recombinant GM-CSF) is not superior to placebo for inducing clinical improvement or remission in active CD (117). Notably, a recent study revealed the complex role of ILC3-derived GM-CSF in the context of intestinal infection and inflammation. GM-CSF can regulate the activation and polarization of intestinal macrophages in humans and mice, driving the differentiation of pro-inflammatory and microbicidal M1 macrophages, while suppressing wound-healing, pro-fibrotic macrophages (69) (**Table 2**). Importantly, compared with “non-complicated” biopsies, biopsies from CD patients with complicated disease (stricturing and penetrating behavior) had enrichment of genes that are upregulated in ILC-depleted macrophages, suggesting that ILC3-derived GM-CSF controls the progression of intestinal fibrosis (69). This is consistent with a study that revealed reduced GM-CSF bioactivity was associated with stricturing/penetrating behavior in CD (114). Thus, the role of ILC3s-derived GM-CSF remains to be elucidated.

The frequency of IL-17-expressing CD56^−^ ILC3s was increased in the inflamed ileum and colon of CD patients compared with non-IBD controls, but not patients with UC (118). IL-17A expression by NCR^−^ ILC3s has been demonstrated to drive colitis development in T-bet^−/−^.Rag2^−/−^ (TRUC) mice (72). Additionally, production of IL-17 and IFN-r in murine ILC3 contributes to colitis development in *H. hepaticus*-mediated innate colitis in mice, and neutralization of IL-17 or INF-r can significantly attenuate colitis (73). Interestingly, Rora^+^ ILC3s result in fibrosis mediated by IL-22 and IL-17 production in a salmonella-induced intestinal fibrosis mouse model, and neutralization of IL-17A can attenuate fibrosis, but the effect of neutralized IL-22 expression was not explored in the study (68) (**Table 2**). However, secukinumab, a human anti-IL-17A monoclonal antibody, failed to show efficacy in CD (119), and this may be due to severe weakening of intestinal epithelial barrier function induced by IL-17 inhibition (120, 121). Additionally, Rag2^−/−^ mice that received T cells from IL17A^−/−^ mice had increased frequency of ILC3, mainly CD4^+^ILC3 and ILC1s as well as enhanced expression of IL-6 and IL-22, which may partly account for the failure of IL-17A inhibitors in CD (122).

Ectopic or tertiary lymphoid tissues (TLTs) are regarded as ectopic clusters of immune cells in response to chronic non-resolving inflammation, and are a pathologic hallmark of CD (123). TLTs have been observed in the mesenteric creeping fat of patients with CD. Furthermore, the formation of functional LTLs in CD-affected mesentery may be attributed to high local levels of CXCL16, CCL20, CCL21, CXCL13, and CCL19, produced by the mesenteric adipocytes (124). It has been reported that human neuropilin-1(NRP1)^+^ LTi-like ILC3s were observed in lung tissues from patients with chronic obstructive pulmonary disease, which may participate in ectopic lymphocyte accumulation (125). Additionally, the frequency of human NKp44^+^ILC3s was significantly reduced in advanced colorectal cancer and non-small cell lung cancer. Furthermore, the accumulation of NKp44^+^ILC3s may be implicated in the formation of tumor-associated TLTs (126, 127). Studies in mice with IL-7 overexpression provide evidence for the indispensable role of LTi cells in the formation of TLTs (128). However, the formation of TLTs was observed in the intestine of RORγt-deficient mice treated with DSS, indicating that TLTs development seems to be independent of LTi cells (129). Collectively, these findings suggest that future work is required to clarify the role of LTi-like ILC3 in the formation of LTLs in both humans and mice. In addition, although the formation of TLTs aggravates colitis in mice (129), the precise role of TLTs in the pathogenesis of IBD must be further explored, and are reviewed in detail elsewhere (123).

Patients with IBD have an increased risk of developing colorectal cancer (CRC). ILC3s and IL-22 seem to play a pathogenic role in the onset or progression of colorectal cancer (14). ILC3s are vital for IL-23-mediated initiation of gut tumorigenesis (130). IL-22 stimulates STAT3 activation in intestinal epithelial cells to promote cell proliferation, playing a predominant role in the maintenance of tumor development (65, 66). Impaired production of IL-22 by ILC3s and insufficient STAT3 activation thereafter account for the protection of *Card9^-/-^* mice from colitis-associated cancer (65). Moreover, NKp46^-^ILC3s drive the transition from colitis to CRC in *Helicobacter hepaticus* (Hh)+AOM mice and neutralization of IL-22 can ameliorate established colitis and reduce tumor burden (66). Besides, the absence of IL-22BP, a neutralizing soluble IL-22 receptor, accelerates tumorigenesis in AMO/DSS treated mice (131). However, IL-22 secretion by ILC3s and γδ T cells regulates the DNA damage response (DDR) in colon stem cells and protects them from acquiring dangerous mutations after genotoxic exposure, thus limiting tumorigenesis (36) (**Table 2**).

## Therapeutic Potential of ILC3s in IBD

Anti-TNF treatment has dramatically improved the treatment of IBD over the past two decades, but primary non-response and secondary loss of response are commonly observed (132). Importantly, anti-TNF agents can result in some adverse events in a fraction of patients (133). Thus, safe and effective therapies for IBD are urgently needed.

IL-23 is a heterodimeric cytokine composed of an IL-23-specific P19 subunit and a P40 subunit shared with IL-12. IL-23 responsive ILC3s participate in the pathogenesis of IBD. Ustekinumab, a monoclonal antibody that targets IL-12/IL-23p40, is effective at inducing and sustaining clinical remission in patients with CD, and has shown some evidence of efficacy in UC patients (132). Moreover, IL-23p19 inhibitors including risankizumab, brazikumab, and mirikizumab have been shown to be effective in patients with moderate-to-severe active CD (134, 135) or UC (136) in clinical studies. However, the influence of IL-23 blockade on ILC3s remains to be elucidated. Compared with placebo, brazikumab can significantly reduce the serum levels of IL-22, and CD patients with baseline serum IL-22 concentration ≥15.6 pg/ml are more likely to experience a clinical response or remission at week 8 following treatment with brazikumab compared with patients with low baseline IL-22 concentration (<15.6 pg/ml) (135).

AHR activation may be a potential therapeutic strategy for the treatment of UC. The AHR pathway mediates crosstalk between particular metabolites in the environment and immune cells, which is important for gut barrier protection and mucosal immunity. I3C can prevent TNBS-induced colitis in mice primarily through inducing IL-22 production by ILC3s (137). Furthermore, fecal microbiota transplantation (FMT) and indigo naturalis (IN), a traditional herbal medicine used for UC, can attenuate DSS-induced colitis in mice by up-regulating the expression or activity of AHR (138, 139). Besides, FMT significantly modulates bacterial metabolism of tryptophan indicated by increased levels of indole-3-acetic acid, which is in line with AHR activation in the colon of recipient piglets (140). A randomized controlled clinical trial showed that 8-week treatment with IN was able to induce clinical responses and mucosal healing in patients with UC. However, the long-term administration of IN should be carefully considered in view of potential adverse effects (141). NPD-0414-2 and NPD-0414-24, novel chemical AHR ligands, up-regulate IL-22 and down-regulate IFN-γ transcripts in LPMCs from IBD patients *in vitro*, which can attenuate TNBS-induced colitis in mice with enhanced expression of IL-22 and reduced expression of IFN-γ in an AHR-dependent manner, without clinical signs of systemic toxicity (142). Of note, PY109, an AHR agonist that has physiochemical drug-likeness properties, ameliorates DSS-induced colitis in mice by promoting the expansion of ILC3s and γδ T cells and expression of IL-22 and IL-17 (143). Collectively, novel AHR agonists with good safety profiles may be effective therapeutic options for the treatment of UC.

Deficiency of Vitamin D is frequently observed in patients with IBD and is associated with increased disease activity and elevated healthcare utilization (144). A prospective study has shown that low serum vitamin D levels (≤ 35 ng/ml) during clinical remission are associated with increased risk of UC relapse (145). In another observational study, CD patients with vitamin D deficiency (25-OH-D concentration < 50 nmol/L) had more relapses during the previous year (146). Importantly, IBD patients with low vitamin D levels who received vitamin D supplements had a significant reduction in their healthcare utilization (144) and correction of 25(OH)D status was associated with reduced risk of CD-related surgery (147). Furthermore, vitamin D status may affect the initial response to TNF inhibitor therapy and IBD patients who had normal vitamin D levels at the initiation of treatment with TNF-α inhibitors had increased odds of remission at 3 months (148). In addition, administration of vitamin A for two months can significantly facilitate clinical remission, clinical response and mucosal healing in UC patients. However, excessive vitamin A supplementation should be avoided due to increased risk of bone fracture and liver toxicity (149). As there are limited data from clinical trials of vitamin D and A in IBD, further studies are needed to conclude whether their administration is clinically effective. In addition, further study to define the optimal levels of vitamin D and A in serum to achieve clinical response is needed.

## Conclusions

ILC3s function as “communication hubs”, which respond to environmental cues and propagate signals to the broader immune system. Herein, we highlighted the dependence of ILC3s on dietary metabolites such as vitamin D, vitamin A as well as its metabolite RA and AHR ligands, microbiota and microbial metabolites such as SCFAs and microbial tryptophan metabolites. In addition, other metabolites such as PGE2 and 7a,25-OHC can also be sensed by ILC3s and give rise to functional outputs. Moreover, maternal nutritional status can modulate ILC3s biology in offspring. Group 3 innate lymphoid cells maintain mucosal homeostasis dependent on moderate production of IL-22, IL-17 and GM-CSF in the steady state. However, excessive production may contribute to the progression of IBD and colorectal cancer. Importantly, IL-22 and IL-17 produced by ILC3s may be involved in the development of intestinal fibrosis. Targeting ILC3s hold promise for treating IBD. Antibodies targeting IL-23-specific P19 or P40 have shown clinical efficacy. In addition, AHR agonist with good safety profiles may be effective treatments for UC. Importantly, nutritional interventions and dietary modifications should always be considered in patients with IBD.

## Author Contributions

DS drafted the initial manuscript. ZR provided critical feedback. All authors contributed to the article and approved the submitted version.

## Funding

This work was supported by the National Natural Science Foundation of China (grant 81670497).

## Conflict of Interest

The authors declare that the research was conducted in the absence of any commercial or financial relationships that could be construed as a potential conflict of interest.
